# The Sensory Abnormality Mediated Partially the Efficacy of Repetitive Transcranial Magnetic Stimulation on Treating Comorbid Sleep Disorder in Autism Spectrum Disorder Children

**DOI:** 10.3389/fpsyt.2021.820598

**Published:** 2022-01-24

**Authors:** Lei Gao, Chen Wang, Xiao-rong Song, Li Tian, Zhi-yi Qu, Yu Han, Xin Zhang

**Affiliations:** ^1^Department of Maternal, Child and Adolescent Health, School of Public Health, Tianjin Medical University, Tianjin, China; ^2^Department of Cerebral Functional Therapy, Tianjin Anding Hospital (Tianjin Mental Health Center), Tianjin, China

**Keywords:** autism spectrum disorder (ASD), repetitive transcranial magnetic stimulation (rTMS), dorsolateral prefrontal cortex (DLPFC), sleep disorders, sensory problems, Bayesian mediation analysis

## Abstract

Sleep disorder emerges as a common comorbidity in children with autism spectrum disorder (ASD), and the interaction between the core symptoms of ASD and its sleep disorder remains unclear. Repetitive transcranial magnetic stimulation (rTMS) was used on the bilateral dorsolateral prefrontal cortex (DLPFC) to investigate the efficacy of rTMS on the core symptoms of ASD and comorbid sleep problems as well as the mediation role of the ASD symptoms between rTMS intervention and sleep improvement. A total of 41 Chinese children with ASD and who met the criteria in the fifth edition of the American Diagnostic and Statistical Manual of Mental Disorders were recruited, and 39 of them (mean age: 9.0 ± 4.4 years old; the male–female ratio was 3.9: 1) completed the study with the stimulating protocol of high frequency on the left DLPFC and low frequency on the right DLPFC. They were all assessed three times (before, at 4 weeks after, and at 8 weeks after the stimulation) by the Children's Sleep Habits Questionnaire (CSHQ), Strengths and Difficulties Questionnaire (SDQ), Childhood Autism Rating Scale, Repetitive Behavior Questionnaire-2, and Short Sensory Profile (SSP). The repeated-measures ANOVA showed that the main effect of “intervention time” of CSHQ (*F* = 25.103, *P* < 0.001), SSP (*F* = 6.345, *P* = 0.003), and SDQ (*F* = 9.975, *P* < 0.001) was statistically significant. By Bayesian mediation analysis, we only found that the total score of SSP mediated the treating efficacy of rTMS on CSHQ (αβ = 5.11 ± 1.51, 95% CI: 2.50–8.41). The percentage of mediation effect in total effect was 37.94%. Our results indicated the treating efficacy of rTMS modulation on bilateral DLPFC for both autistic symptoms and sleep disturbances. The sensory abnormality of ASD mediated the improvement of rTMS on sleep problems of ASD.

## Introduction

Autism spectrum disorder (ASD) is a severe neurodevelopmental disorder characterized by social–communicative atypicality and a restrictive and rigid repertoire of behaviors (1). Over the past 30 years, the prevalence of ASD has dramatically increased. In the United States, around one out of 59 children have been diagnosed with ASD (2). Cumulative evidence has shown that as many as 80% of children with ASD suffered from a variety of sleep problems (3), such as insomnia (4), daytime sleepiness, sleep-disordered breathing, and parasomnias (5), which is much higher than that in typically developing children (6–8). The core symptoms of ASD and their comorbid sleep disturbances interact severely with each other. The predisposition of neurophysiology and neurochemistry possibly makes autistic patients susceptible to chronic sleep–wake disorder (9)—for example, some genes (such as PER1, PER2, and NPAS2) contribute to the sleep–wake regulation processes and ASD (9). At the same time, the symptoms of ASD, such as intolerance to change and persistence in the same pattern, also make autistic children more intolerant of the sleeping environment and have more difficulty in falling asleep (10, 11). On the other hand, the sleep problems exacerbate the severity of the core ASD symptoms, such as repetitive behaviors, social, and communication difficulties (12, 13), and other maladaptive behaviors (14, 15). Exploring the interaction between them can not only help us to understand the etiology of ASD but also provide more information on effective intervention.

Recent work suggests that repetitive transcranial magnetic stimulation (rTMS) has been a promising treatment intervention for both the core symptoms of ASD (such as repetitive and stereotyped behaviors and social behavior) and sleep disturbances (16–20). However, there was no suitable stimulating protocol for both at the same time. Our previous research compared the effects of different rTMS stimulation protocols on the bilateral dorsolateral prefrontal cortex (DLPFC) for ASD. We found that high-frequency left and low-frequency right rTMS can effectively control the core symptoms of autism, especially on the improvement of the anxiety of ASD children (21). Since anxiety is closely related to sleep disorders in children and adolescents (22), some studies have confirmed that ASD symptoms and anxiety uniquely contributed to the presence and frequency of sleep problems (23). Thus, the rTMS protocol of high-frequency left and low-frequency right is supposed to work for both the core autistic symptoms and sleep disturbances.

In the current study, rTMS was applied to the left and right DLPFC with different frequencies (high-frequency left and low-frequency right) to explore the relationship between sleep profiles and core symptoms in ASD at the same time. We hypothesized that both the core symptoms and the sleep problems would be ameliorated by rTMS in ASD, and the improvement of the core symptoms would mediate the promotion of the sleeping situations of ASD children, by which we can offer (a) the verification of an rTMS protocol for both the core symptoms and the comorbid sleep problems and (b) understanding of the role of the core symptoms in the comorbid sleep problems of ASD.

## Methods

### Participants

We released the recruitment information through the outpatient clinic of Tianjin Anding Hospital (the psychiatric hospital) and by online platform from October 2018 to October 2020 to recruit ASD children. The inclusion criteria were as follows: (1) it meets the diagnostic criteria of ASD in the fifth edition of the American Diagnostic and Statistical Manual of Mental Disorders, (2) age of 2–18 years old, (3) no medication during the rTMS intervention, (4) right-handed, and (5) total score of Children's Sleep Habits Questionnaire (CSHQ) at baseline ≥41. The exclusion criteria were as follows (21, 24): (1) contraindications to rTMS, such as metal or electronic instruments near the coil stimulation site; participants with a history of epilepsy (excluding epilepsy according to their electroencephalogram and medical record); participants with a history of brain trauma, brain tumors, and other diseases; participants with severe or recent heart disease or other major physical illnesses; (2) diagnosis of other mental illnesses (e.g., attention-deficit hyperactivity disorder, schizophrenia, and depression); and (3) other neurodevelopmental disorder, genetic metabolic disease, or severe neurological disease. The study was conducted under the Code of Ethics of the World Medical Association (Declaration of Helsinki). The study also complied with all relevant national regulations and institutional policies and had been approved by the Medical Ethics Committee of Tianjin Medical University. The participants and their parents (or legal guardians) obtained all information about the research, including the purpose, requirements, responsibilities, compensation, risks, benefits, and alternatives. All questions were answered before asking for their consent signature.

### TMS Procedure

A trained electrophysiologist delivered rTMS stimulation over the cortical area, controlling the contralateral first dorsal interosseous (FDI) using a magnetic field stimulator (CCY-1, Yiruide Medical Corporation, Wuhan, China) to detect the resting motor threshold (MT). The MT was determined for each hemisphere in all individuals by gradually increasing the output of the machine by 5% until a 5-mV deflection or a visible twitch in the FDI muscle was identified in two out of three trials (24). Electromyographic responses were monitored continuously from the hand contralateral to the stimulated hemisphere using the MEP module in the magnetic stimulator (Yiruide Medical Corporation, Wuhan, China). The subjects were familiarized with the laboratory and the procedure before the first transcranial magnetic stimulation session.

In this study, rTMS was selected to stimulate the left DLPFC with a high frequency (10 Hz) and the right DLPFC with a low frequency (1 Hz) based on the evidence-based basis proposed by the European Union of Neurological Societies (25), and the electrode positioning cap was used for accurate positioning. The specific parameters are as follows: the stimulation frequency of the right dorsolateral prefrontal lobe is 1 Hz, the stimulation time is 32 s, the stimulation number is 32, the intermittent time is 1 s, the repetition number is 28, and the stimulation intensity is 25% MT, whereas the stimulation frequency of the left dorsolateral prefrontal lobe is 10 Hz, the stimulation time is 3.2 s, the stimulation number is 32, the intermittent time is 10 s, the repetition number is 45, and the stimulation intensity is 25% MT. The intervention time of rTMS was five times/week, and the course of intervention was every 4 weeks. Each child had two courses of intervention, for a total of 8 weeks.

### Assessment of the Symptoms of ASD

We used the CSHQ to evaluate the sleep quality of all participants in the current study. The caregivers were required to fill in the CSHQ according to the sleep status of the participants in a typical week in the past month (26). The participants are regarded as having poor sleep quality, with a total score of CSHQ >41. Meanwhile, the Repetitive Behavior Questionnaire (RBQ-2), the Short Sensory Profile (SSP), and the Strengths and Difficulties Questionnaire (SDQ) were used to evaluate the severity of the symptoms of repetitive behaviors, abnormal responses to sensory stimuli, and social impairments in ASD children, respectively. The Peabody Picture Vocabulary Test (PPVT) was also used to evaluate the non-verbal intelligence of autistic children. All the participants were assessed before, after one intervention course, and after two intervention courses.

### Statistical Data Analysis

Statistical analyses were performed on the subject-averaged behavioral data. The primary analysis model was repeated-measures ANOVA (RM-ANOVA), with the following dependent variables: scores of CSHQ, RBQ-2, SSP, and SDQ. Repeated within-group factor was time (*T*_0_ = before rTMS intervention, *T*_1_ = after one course of rTMS intervention, and *T*_2_ = after two courses of rTMS intervention). According to our previous research (25), the sample size was estimated by PASS15.05.

For the mediation analysis, a model of Bayesian mediation analysis was created, with time as the independent variable (time = 0, 1, and 2 as “before rTMS intervention”, “after one course of rTMS intervention,” and “after two courses of rTMS intervention,” respectively), the scores of RBQ-2, SSP, and SDQ as the mediator, respectively, and the score of CSHQ as the dependent variable by using the procedure of MCMC of SAS 9.4 (27).

## Results

A total of 41 ASD children were recruited, of whom two withdrew during the intervention, and 39 ASD children who completed all the intervention and assessment items were finally included in the study. All the participants were right-handed. The average age of the subjects was 9.0 ± 4.4 years old, the male–female ratio was 3.9:1, and the median PPVT was 40.00 (Q1 = 0.00, Q3 = 75.50). Data on age, IQ, severity of autism symptoms, family income, and maternal education level of ASD children are shown in Table 1.

**Table 1:** Demographic characteristics of autism spectrum disorder children (*n* = 39).

**Variables**	***N*** **(%)**
**Gender**	
Male	31 (79.5%)
Female	8 (20.5%)
**Age**	
2.0–5.9	9 (23.1%)
6.0–11.9	21 (53.8%)
12.0–18.0	9 (32.1%)
**Score of PPVT**	
<70	26 (66.7%)
≥70	13 (33.3%)
**Score of CARS**	
<36	22 (56.4%)
≥36	17 (43.6%)
**Monthly household income (RMB: Yuan)**	
<5,000	6 (15.4%)
5,000–7,999	10 (25.6%)
8,000–9,999	6 (15.4%)
≥10,000	17 (43.6%)
**Maternal education**	
College degree or above	27 (69.2%)
High school, technical secondary school, or vocational school	7 (17.9%)
Junior high school and below	5 (12.8%)

*PPVT, Peabody Picture Vocabulary Test; CARS, Childhood Autism Rating Scale*.

### The Results of CSHQ, RBQ-2, SSP, and SDQ After Intervention

For CSHQ, RM-ANOVA revealed that the main effect of “time” was significant (*F* = 25.103, *P* < 0.001): for the total score, the mean was 53.23 ± 6.14, 48.64 ± 5.95, and 46.97 ± 6.18, respectively; and the changes between different time points were all statistically significant (see Table 2). For each subscale, there was a statistical decrease after one or two courses of intervention than before, except for sleep-disordered breathing and sleep-onset delay. For details, there was a significant reduction in sleep duration at the second (*T*_0_ vs. *T*_1_) and third (*T*_0_ vs. *T*_2_) assessments. Similarly, there was a significant reduction in bedtime resistance, sleep anxiety, and daytime sleepiness after one intervention course of rTMS (*T*_0_ vs. *T*_1_), except parasomnias and night wakings, for which a significant reduction was observed only after two intervention courses (see Table 2). However, there was no reduction at all in sleep-disordered breathing and sleep-onset delay.

**Table 2:** Comparison of CSHQ before and after intervention in children with autism spectrum disorder (*n* = 39).

**Items**	**Time^a^**	**Mean difference**	**Standard error**	***P***
Total score	T0–T1	4.59	0.85	0.000
	T0–T2	6.26	0.98	0.000
	T1–T2	1.67	0.56	0.005
Bedtime resistance	T0–T1	1.18	0.25	0.000
	T0–T2	1.33	0.35	0.001
	T1–T2	0.15	0.25	0.547
Sleep anxiety	T0–T1	0.64	0.20	0.003
	T0–T2	0.85	0.24	0.001
	T1–T2	0.21	0.14	0.146
Sleep duration	T0–T1	0.67	0.20	0.002
	T0–T2	1.26	0.29	0.000
	T1–T2	0.59	0.25	0.021
Sleep-disordered breathing	T0–T1	0.10	0.08	0.210
	T0–T2	0.13	0.07	0.058
	T1–T2	0.03	0.05	0.570
Parasomnias	T0–T1	0.33	0.19	0.085
	T0–T2	0.54	0.16	0.002
	T1–T2	0.21	0.12	0.088
Daytime sleepiness	T0–T1	1.23	0.50	0.019
	T0–T2	1.59	0.52	0.004
	T1–T2	0.36	0.44	0.421
Night wakings	T0–T1	0.33	0.17	0.057
	T0–T2	0.39	0.18	0.034
	T1–T2	0.05	0.08	0.534
Sleep-onset delay	T0–T1	0.10	0.08	0.210
	T0–T2	0.15	0.12	0.205
	T1–T2	0.05	0.10	0.599

*T0, before the intervention; T1, after one intervention course; T2, after two intervention courses*.

*CSHQ, Children's Sleep Habits Questionnaire*.

a *Time refers to before, after one intervention course, or after two intervention courses*.

For RBQ-2, the main effect of “time” was not significant either in the total score (*F* = 2.425, *P* = 0.093) or in the subscales. For SSP, the main effect of “time” was significant (*F* = 6.345, *P* = 0.003), which indicated that the total score of SSP reduced significantly after rTMS intervention. For the score of the SSP subscales, there was a significant reduction in tactile sensitivity and auditory filtering, but no such significant changes were observed for the rest of the subscales (see Table 3). For SDQ, the main effect of “time” of the total score was also statistically significant (*F* = 9.975, *P* < 0.001), and the substantial improvement emerged at the second (*T*_0_ vs. *T*_1_) and the third (*T*_0_ vs. *T*_2_) assessment, but with no difference between the second and the third assessment (*T*_1_ vs. *T*_2_). For the subscales of SDQ, there was a significant reduction at the third assessment (*T*_0_ vs. *T*_2_), except for the hyperactivity and inattention as well as peers/peered problems subscales, which had no change throughout the intervention (see Table 3).

**Table 3:** Comparison of SSP and SDQ before and after intervention in children with autism spectrum disorder (*n* = 39).

**Subscales**	**Time^a^**	**Mean difference**	**Standard error**	***P***
**SSP**				
Tactile sensitivity	T0–T1	1.51	0.61	0.017
	T0–T2	1.82	0.57	0.003
	T1–T2	0.31	0.47	0.520
Auditory filtering	T0–T1	1.28	0.63	0.048
	T0–T2	2.18	0.77	0.007
	T1–T2	0.90	0.58	0.130
Total score	T0–T1	4.88	2.31	0.042
	T0–T2	6.52	2.31	0.017
	T1–T2	1.64	2.31	0.825
**SDQ**				
Emotional problems	T0–T1	0.73	0.47	0.323
	T0–T2	1.30	0.47	0.018
	T1–T2	0.58	0.47	0.523
Conduct problems	T0–T1	0.72	0.38	0.159
	T0–T2	0.93	0.38	0.045
	T1–T2	0.20	0.38	0.934
Prosocial	T0–T1	1.00	0.54	0.192
	T0–T2	1.48	0.54	0.023
	T1–T2	0.48	0.54	0.767
Total score	T0–T1	2.65	1.08	0.047
	T0–T2	4.83	1.08	<0.001
	T1–T2	2.18	1.08	0.446

*T0, before the intervention; T1, after one intervention course; T2, after two intervention courses*.

*SSP, Short Sensory Profile; SDQ, Strengths and Difficulties Questionnaire*.

a *Time refers to before, after one intervention course, or after two intervention courses*.

### The Result of the Bayesian Mediation Analysis

**Figure 1:**
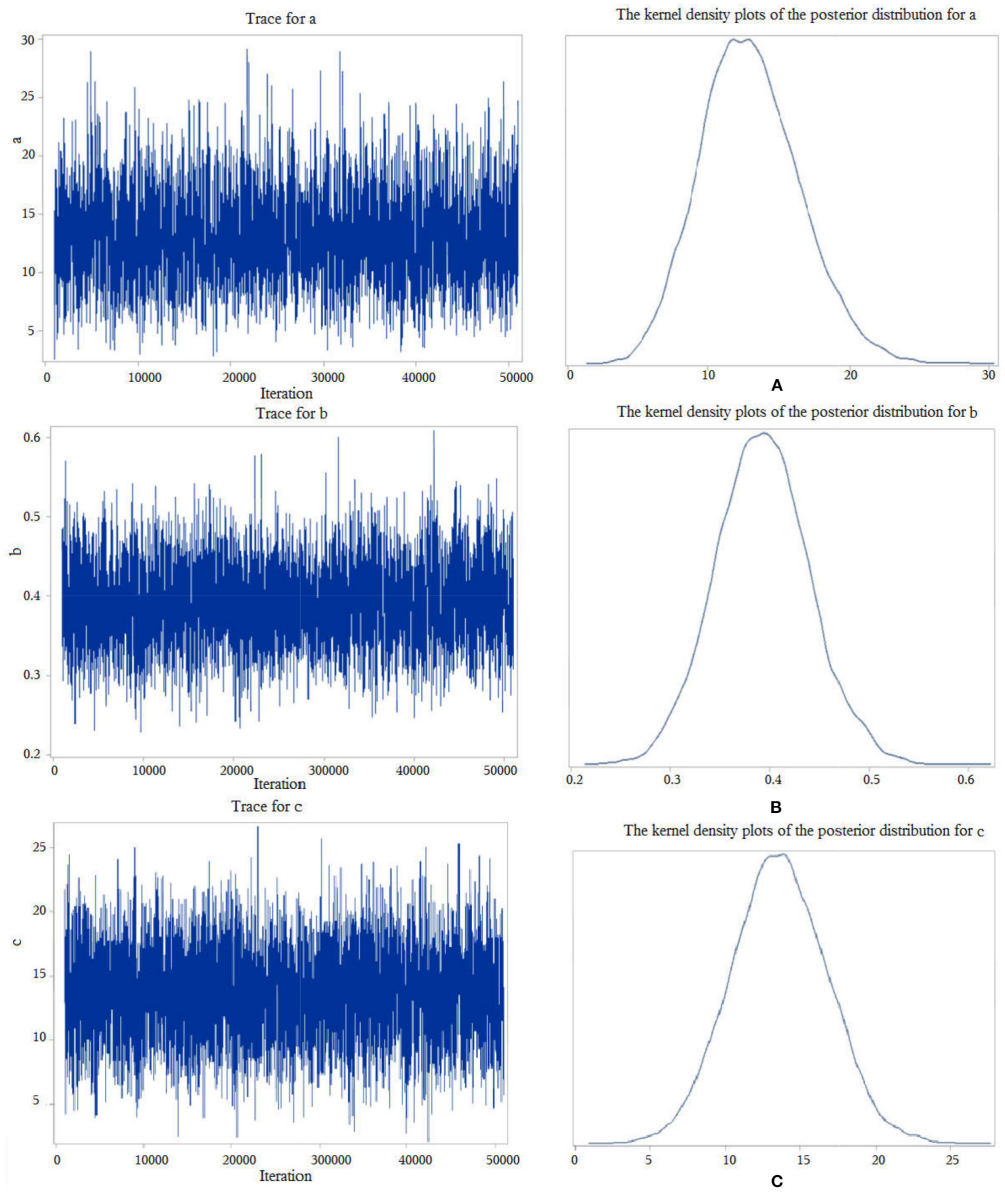
Trace plots and kernel density plots of the posterior distribution for the parameters. “c” represents the total effect of the independent variable X on the dependent variable Y, “b” measures the relation between the mediator M and the dependent variable Y, adjusted for the independent variable X, and “a” measures the relation between X and M.

First, we performed a Bayesian mediation analysis with the total score of SSP mediating the relationship between rTMS intervention and the total score of CSHQ. The trace plots indicated a good mixing for the parameters, and the chains that mix well tend to converge sooner (see Figure 1), as well as the kernel density plots of the posterior distribution for the given parameter (see Figure 1). The posterior mean of the mediated effect of rTMS intervention through the total score of SSP on change in the total score of CSHQ was αβ = 5.11 ± 1.51, with a 95% central credibility interval of 2.50–8.41. Given that 0 was not between the two credibility limits, the mediated effect of SSP was statistically significant. A further calculation (αβ / c × 100%) reflected that the mediation effect was 37.94%, which means that 37.94% of the total effect between rTMS intervention and the score of CSHQ was mediated by the score of SSP (see Table 4). Our results indicated that rTMS intervention could promote the sleep of autistic children through improving the sensory abnormalities in addition to the direct reduction of the sleep problems. However, neither the total score of SDQ nor of RBQ-2 was the mediator due to the poor convergence of the Markov chain or the containment of 0 in the 95% central credibility interval, which meant that the mediated effect is not statistically significant.

**Table 4:** Parameter summary of Bayesian mediation analysis with SSP as mediator.

**Parameter**	***N***	**Mean**	**Standard deviation**	**95% CI of highest posterior** **density**	
				**Lower**	**Upper**
α	10,000	13.01	3.50	6.41	19.96
β	10,000	0.39	0.05	0.30	0.48
c	10,000	13.63	3.18	7.20	19.57
H	10,000	−0.19	0.04	−0.27	−0.12
αβ	10,000	5.11	1.51	2.50	8.41

*“c” represents the total effect of independent variable X on the dependent variable Y, “αβ” represents the effect of X on Y, adjusted for the effect of the mediator M, “β” measures the relation between the mediator M and the dependent variable Y, adjusted for the independent variable X, and “α” measures the relation between X and M*.

*SSP, Short Sensory Profile*.

## Discussion

As mentioned in the “Introduction,” the high prevalence of sleep problems in children with ASD is more likely to be attributed to the pathogenesis of ASD, such as brain wave organizational and maturational differences, arousal and sensory dysregulation, circadian-relevant genes, and abnormal melatonin production (28, 29). However, there is no intervention especially for the sleep problems of autistic individuals besides the conventional measures aimed at the behavioral insomnia of children caused by external factors, such as sleep education, sleep environment improvement, behavioral intervention, and drug treatment (such as exogenous melatonin) (28, 30). Therefore, the intervention on the sleep problems in autistic individuals should consider the treatment of the core symptoms and sleep problems (31). The current study examined the efficacy of the rTMS protocol (high frequency on the left + low frequency on the right) on bilateral DLPFC for both the core symptoms (such as social impairments, repetitive behaviors, and sensory abnormalities) and sleep problems in ASD youth. Our results revealed that both the core symptoms (such as tactile and auditory abnormalities, emotional, conduct, and social problems) and sleep problems improve significantly after two courses of intervention of rTMS. Our study provides new ideas for the intervention of sleep problems in ASD.

Previous studies have shown the efficacy of rTMS on either ASD symptoms, such as repetitive and stereotyped behaviors and social behavior (18, 32), or chronic primary insomnia, such as reducing sleep latency and increasing total sleep time and rapid eye movement latency (33, 34). However, the research on the treatment of the sleep problems of ASD individuals is far from enough. To our knowledge, the rTMS protocol for ASD comorbid sleep disturbances has not been reported yet. One of the reasons is that we lack the proper protocols for ASD symptoms and sleep problems—for example, most of the studies of rTMS for ASD symptoms chose the DLPFC for stimulating the location, but with varied protocols (35): for example, the high-frequency (10 Hz) stimulation on the left DLPFC only (36), the low-frequency rTMS on the left DLPFC (37), or the low-frequency stimulation on bilateral DLPFC (38, 39). Similarly, the rTMS protocols on sleep problems are also full of inconsistencies—for example, in terms of insomnia, most studies used the protocol of low frequency (1 Hz) on the right DLPFC (40); some researchers also chose the non-DLPFC targets to treat patients with sleep problems (41). In the current study, the high-frequency rTMS on the left DLPFC and the low-frequency rTMS on the right DLPFC worked for both ASD symptoms and sleep-related problems. The partial sharing pathogenesis of both the core symptoms and comorbid sleep problems of ASD offered the possibility of intervention of rTMS for both with the same protocol—for example, the cortical excitation/inhibition imbalances and the abnormal excitatory–inhibitory ratio in ASD patients (42, 43). The high-frequency rTMS stimulation of the left DLPFC could cause a long-term potentiation (LTP) of synaptic transmission in the stimulation area (44), and LTP could spread to the cortex and the subcortical neural network (45, 46), which led to the enhancement of excitability of the mirror neuron system in ASD patients, to improve the understanding of the social environment in ASD patients and enhance the ability of imitation (47). Thus, it finally improved the core symptoms of ASD patients. The high-frequency rTMS stimulation of the left DLPFC could also improve the sleep problems of ASD individuals by improving their arousal dysregulation and the dysregulation of the autonomic nervous system (28, 48, 49). In addition, the low-frequency rTMS of the right DLPFC could improve the core symptoms of ASD individuals by activating the inhibitory GABAergic double-bouquet interneurons to improve the excitation/inhibition balance of the prefrontal cortex in patients with ASD (50). In addition, the low-frequency rTMS on the right DLPFC could improve sleep by increasing the serum brain-derived neurotrophic factor and γ aminobutyric acid concentration and reducing motor-evoked potentials (18).

Similarly, the sharing abnormality of the gamma frequency oscillations probably interpreted the mediation effect of the sensory problems in ASD youth between the rTMS intervention and improvement of sleep. It is believed that the cortical gamma-band activity participates in a variety of mental activities, including sensory perception (51); at the same time, the gamma oscillation is the representation of the rhythm of wakefulness and sleep (52). The improvement of gamma oscillation seems to be the potential mechanism of the sensory profile of ASD children in the amelioration of their sleep disturbances by rTMS. The present findings offer a further understanding of the pathogenesis and therapeutic prospect of sleep problems in autistic patients.

There are some limitations in our study; we failed to access the sleep problems of autistic children with more objective measurements, such as by polysomnography (PSG), due to the severe discomfort caused by PSG measurement and the low tolerance of the autistic subjects. In addition, the sample size in the current research is relatively small, although the sample size calculated based on our previous study is enough. We did not try other rTMS protocols (different stimulation locations) due to the limitation of time and research funds. Future research on neuroimaging, such as functional magnetic resonance imaging or functional near-infrared spectroscopy, might extend the explanations of the abnormality of the DLPFC of ASD youth comorbid with sleep disturbances.

## Data Availability Statement

The datasets presented in this article are not readily available because their containing information in the data probably compromise the privacy of research participants. Requests to access the datasets should be directed to Xin Zhang, zhangxin@tmu.edu.cn.

## Ethics Statement

The studies involving human participants were reviewed and approved by the Medical Ethics Committee of Tianjin Medical University. Written informed consent to participate in this study was provided by the participants' legal guardian/next of kin.

## Author Contributions

XZ and LG obtained funding, had full access to all the data in the study, and take responsibility for the integrity of the data and the accuracy of the data analysis. XZ, LG, and LT contributed to the conception and design of the study. XZ, LG, YH, Z-yQ, X-rS, and CW contributed to the acquisition, analysis, or interpretation of data. LG performed the statistical analysis. LG, LT, and XZ wrote the first draft of the manuscript. All authors contributed to the article and approved the submitted version.

## Funding

This study has received research grants from National Natural Science Foundation of China (Grant Nos. 81673200 and 81973070). The funders of the study had no role in the design and conduct of the study; collection, management, analysis, and interpretation of the data; and preparation, review, or approval of the manuscript or the decision to submit for publication.

## Conflict of Interest

The authors declare that the research was conducted in the absence of any commercial or financial relationships that could be construed as a potential conflict of interest.

## Publisher's Note

All claims expressed in this article are solely those of the authors and do not necessarily represent those of their affiliated organizations, or those of the publisher, the editors and the reviewers. Any product that may be evaluated in this article, or claim that may be made by its manufacturer, is not guaranteed or endorsed by the publisher.
